# Mental Health Surveillance am Robert Koch-Institut – Strategien zur Beobachtung der psychischen Gesundheit der Bevölkerung

**DOI:** 10.1007/s00103-023-03678-4

**Published:** 2023-02-27

**Authors:** Julia Thom, Lena Walther, Sophie Eicher, Heike Hölling, Stephan Junker, Diana Peitz, Julia Wilhelm, Elvira Mauz

**Affiliations:** Abteilung für Epidemiologie und Gesundheitsmonitoring, Robert Koch-Institut, Berlin, Deutschland

**Keywords:** Mental Health Surveillance, Public Health, Psychische Gesundheit, Allgemeinbevölkerung, COVID-19-Pandemie, Mental health surveillance, Public health, Mental health, General population, COVID-19 pandemic

## Abstract

Die fortlaufende und systematische Beobachtung der Bevölkerungsgesundheit ist grundlegend für eine effektive Public-Health-Praxis. Angesichts der wachsenden Bedeutung psychischer Gesundheit für die Bevölkerungsgesundheit wird am Robert Koch-Institut eine Mental Health Surveillance für Deutschland aufgebaut. Deren Ziel ist es, kontinuierlich verlässliche Einschätzungen zur psychischen Gesundheit der Bevölkerung und ihrer Entwicklung zur Verfügung zu stellen.

Aktuell werden 3 Surveillance-Strategien verfolgt: 1) Regelmäßige Bestandsaufnahmen sollen den psychischen Gesundheitszustand der Bevölkerung über eine Vielfalt an Indikatoren und Datengrundlagen umfassend beschreiben und langfristige Veränderungen aufzeigen. Sie knüpfen an bereits vorliegende Arbeiten aus Epidemiologie und Versorgungsforschung an. 2) Die hochfrequente Beobachtung einer Auswahl an Indikatoren dient der frühen Erkennung von Trends. 3) Das kontinuierliche Literaturreview erfasst monatlich die aktuelle Evidenzlage zur Entwicklung der psychischen Gesundheit in der COVID-19-Pandemie. Die beiden letzten Strategien entstanden in Reaktion auf veränderte Informationsbedarfe während der Pandemie.

In diesem Beitrag werden die 3 Strategien, ihre Funktionen, Grenzen und Entwicklungspotenziale beschrieben und diskutiert. Vermittelt über verschiedene Formate der Berichterstattung können sie Handlungs- und Forschungsbedarfe im Bereich Public Mental Health aufzeigen. Bei weiterem Aufbau und Betrieb hat Mental Health Surveillance insgesamt das Potenzial, die Erreichung von Zielsetzungen im Bereich Public Mental Health zu unterstützen und auf verschiedenen Ebenen zur Verbesserung der gesundheitlichen Lage beizutragen.

## Abstract (en)

The continuous and systematic surveillance of the health of populations is fundamental for effective public health practice. In light of the growing importance of mental health within population health, a Mental Health Surveillance for Germany is being established at the Robert Koch Institute. Its aim is to continually provide reliable information on the current state and development of the mental health of the population.

Three surveillance strategies are currently being pursued: 1) Regular comprehensive assessments aim to describe the mental health status of the population using a wide range of indicators and data sources and to observe long-term developments. They build on existing work in epidemiology and health services research. 2) High-frequency monitoring of a selection of indicators is used for the early detection of trends. 3) A continuous literature review collates current findings on mental health developments in the COVID-19 pandemic on a monthly basis. The latter two strategies were implemented in response to new information needs in the pandemic.

This paper describes and discusses these three strategies and their functions, limitations, and potential for development. Their results are communicated through different forms of reporting and serve to identify needs for action and research in public mental health. The further development and long-term operation of the Mental Health Surveillance as a whole has the potential to facilitate the achievement of public mental health objectives and to contribute on different levels to the improvement of population health.

## Hintergrund und Aufbau einer Mental Health Surveillance

### Public Health Surveillance

Die Bedeutung von Public Health Surveillance (PHS) für den Gesundheitsschutz der Bevölkerung ist durch die COVID-19-Pandemie eindrücklich deutlich geworden. Fehlte es an einer verlässlichen Datenlage zum Infektionsgeschehen, wurde vor „Blindflug“ [1] gewarnt. Um diesen grundsätzlich zu vermeiden, zählt PHS zu den 10 Eckpunkten einer Public-Health-Strategie für Deutschland [2].

PHS umfasst eine fortlaufende und systematische Erhebung, Zusammenführung, Analyse, Interpretation und zeitnahe Dissemination von Daten zu zentralen Themen der Bevölkerungsgesundheit [3]. In Abgrenzung zu epidemiologischer Forschung dient Surveillance primär der Beschreibung gesundheitlicher Entwicklungen in der Bevölkerung und nicht ihrer Erklärung. Ergebnisse von Surveillance tragen vielmehr dazu bei, Bedarfe für Forschung und Public-Health-Maßnahmen zu erkennen. Der Ansatz sieht zudem vor, dass Ergebnisse an Akteure aus der Public-Health-Praxis kommuniziert werden und nicht allein akademischen Zwecken dienen. Mental Health Surveillance (MHS) stellt damit eine Perspektive von Public Mental Health (PMH) dar, die auf die Berichterstattung zur psychischen Bevölkerungsgesundheit (Population Mental Health) fokussiert.

Psychische Gesundheit wird erst in jüngerer Vergangenheit im Surveillance-Paradigma betrachtet [4]. Noch fehlt es in vielen Ländern am nötigen Berichtswesen, weshalb der Mental Health Action Plan (2013–2030) der Weltgesundheitsorganisation (WHO) fordert, nationale Informationssysteme für Mental-Health-Indikatoren aufzubauen [5]. Diese Forderung nach MHS wurde durch die disruptiven Effekte der COVID-19-Pandemie auf die psychische Gesundheit der Bevölkerung verstärkt [6, 7].

### Aufbau von Mental Health Surveillance für Deutschland am Robert Koch-Institut

2018 beauftragte das Bundesgesundheitsministerium (BMG) das Robert Koch-Institut (RKI) mit dem Aufbau einer MHS für Deutschland. Ausgangspunkt waren die bis dato fragmentierte Datenlage, Beispiele von MHS in anderen Ländern (ausführliche Darstellung siehe [8]) sowie die Ergebnisse einer Recherche zu etablierten Indikatoren psychischer Gesundheit [9].

Unter Einbezug internationaler Expertise [10] wurde ein Konsentierungsprozess mit nationalen Stakeholdern durchgeführt [8]. Dieser resultierte in einer Auswahl von 60 Indikatoren, die ein PMH-Rahmenkonzept abbilden. Es umfasst Determinanten psychischer Gesundheit, Merkmale des psychischen Gesundheitszustandes und der Versorgung psychischer Störungen sowie Kennwerte von Krankheitslast und Teilhabe. Einbezogen wurden Indikatoren, die sowohl inhaltlich bedeutsam als auch prinzipiell durch Maßnahmen veränderbar sind (im Gegensatz zu weniger beeinflussbaren Merkmalen wie Persönlichkeit). Das Rahmenkonzept bildet die Grundlage für den Aufbau einer systematischen Berichterstattung, die bisherige Evidenzlücken schließen soll.

Während der Konzeption des MHS-Systems am RKI brachte die COVID-19-Pandemie neue Informationsbedarfe mit sich, die in der empirischen Umsetzung berücksichtigt wurden. Zum aktuellen Stand werden 3 MHS-Strategien mit jeweils spezifischer Funktion kombiniert: regelmäßige Bestandsaufnahmen, hochfrequente Beobachtung und kontinuierliches Literaturreview (Tab. 1). Diese Strategien unterscheiden sich in Zielsetzung und Fragestellung, untersuchten Indikatoren und Datengrundlagen sowie Schwerpunktsetzungen der Datenanalyse, Berichterstattung und Interpretation.

**Table 1:** 

	Regelmäßige Bestandsaufnahmen	Hochfrequente Beobachtung	Kontinuierliches Literaturreview
*Ziele*	Psychischen Gesundheitszustand der Bevölkerung langfristig regelmäßig und umfassend beschreiben, zeitliche Entwicklungen aussagekräftig abbilden	Aktuelle Entwicklungen kontinuierlich beobachten, Veränderungen frühzeitig erkennen	Aktuelle Forschungsaktivitäten und Evidenz zur Entwicklung der psychischen Gesundheit der Bevölkerung (in der COVID-19-Pandemie) regelmäßig erfassen
*Frequenz der Beobachtung*	Im Abstand mehrerer Jahre	Engmaschig, z. B. monatlich oder quartalsweise	Je nach Informationsbedarfen und Publikationsaufkommen, z. B. monatlich
*Zweck*	Langfristig Gesamtbild zeitlicher Entwicklungen psychischer Gesundheit bereitstellen	Frühwarnsystem, Krisenbereitschaft sicherstellen, in Krisen evidenzbasierte Response ermöglichen	Evidenzlage sondieren und zusammenfassen
*Indikatoren*	Breites Spektrum an Indikatoren zur Abbildung von Public-Mental-Health-Rahmenkonzept	Auswahl von Indikatoren zur Entdeckung von Veränderungen	Abhängig von publizierten Ergebnissen, weitgehend offene Suchstrategie
*Datengrundlagen*	Befragungs- und Routinedaten, Triangulation zwischen Datenquellen	Kontinuierlich und zeitlich engmaschig erhobene/ausgewertete Befragungs- und Routinedaten	Literaturdatenbanken, ausgewählte Quellen für Handrecherche
*Fragestellungen*	– Wie häufig sind zentrale Merkmale der psychischen Gesundheit in der Bevölkerung? – Wie verteilen sie sich zwischen verschiedenen Bevölkerungsgruppen? – Welche Entwicklungen zeigen Häufigkeiten und Verteilungen langfristig? – Inwiefern zeigen sich bei verschiedenen Merkmalen ähnliche oder divergierende Entwicklungen?	– Deuten jüngste Entwicklungen vor dem Hintergrund des Gesamtverlaufs auf eine Verschlechterung, Verbesserung oder Stabilität der psychischen Gesundheit in der Bevölkerung hin? – Welche Entwicklungen zeigen die Merkmale über die Zeit und koinzident mit kollektiven Ereignissen oder (gesundheits‑)politischen Interventionen?	– Für welche Beobachtungszeiträume und Indikatoren liegen veröffentlichte Ergebnisse zur Entwicklung psychischer Gesundheit in der Allgemeinbevölkerung (während der COVID-19-Pandemie) vor? – Wie verlässlich sind Daten und Ergebnisse? – Wie ist die vorliegende Evidenz zusammenfassend zu bewerten?

Ziel des Beitrages ist es, diese 3 MHS-Strategien anhand von empirischen Beispielen zu beschreiben sowie ihre Funktionen, Grenzen und Entwicklungspotenziale zu diskutieren.

## Regelmäßige Bestandsaufnahmen: Status und Entwicklung psychischer Gesundheit der Bevölkerung

### Ausgangslage

Beschreibungen der psychischen Gesundheit der Bevölkerung stützen sich bislang auf Ergebnisse aus Gesundheitsmonitoring, Epidemiologie und Versorgungsforschung, die zwar in vielfältiger Form vorliegen, aber fragmentiert sind. Diese bisher lückenhafte Datenlage erlaubt weder verlässliche Aussagen noch einen konzisen Überblick über zentrale Entwicklungen, was auch in anderen Ländern als Herausforderung beschrieben wird [11, 12].

Übersichtsarbeiten aus Deutschland ziehen verschiedenste und teils veraltete Belege der Public-Health-Relevanz psychischer Gesundheit heran [13–18], wie folgende Beispiele illustrieren: Als aktuellster Messwert der Prävalenz psychischer Störungen in der Bevölkerung wird auch im Jahr 2022 noch der Wert von 27,8 % aus den Erhebungsjahren 2009–2012 angegeben [19]. Mortalität wurde bisher nur für eine Auswahl psychischer Störungen im Jahr 2012 untersucht, wo sich eine Verkürzung der Lebensdauer von bis zu 12 Jahren zeigte [20]. Als Belege der starken Einschränkung von Lebensqualität und Funktionsfähigkeit, zu der psychische Störungen führen, werden anstelle von bevölkerungsbezogenen Daten Werte einzelner Krankenkassen herangezogen [21, 22]. Auch die Entwicklung der Krankheitskosten psychischer Störungen kann bisher nicht sicher eingeschätzt werden, da Ergebnisse nur für einzelne Jahre berichtet wurden, zuletzt für 2020 mit 56 Mrd. €, was rund 13 % aller Krankheitskosten entspricht [23].

Ausgehend von diesem Mangel an fortlaufender Evidenz zu Kernindikatoren psychischer Bevölkerungsgesundheit greift die Strategie regelmäßiger Bestandsaufnahmen PHS-Bedarfe auf, die bereits vor der Pandemie bestanden, und orientiert sich daher am bis dato etablierten Anspruch an PHS im Bereich nichtübertragbarer Erkrankungen.

### Ziele und Fragestellungen

Ziel regelmäßiger Bestandsaufnahmen zur psychischen Gesundheit ist es, deren Zustand langfristig regelmäßig und umfassend zu beschreiben und so zeitliche Entwicklungen über Jahre und Jahrzehnte aussagekräftig abzubilden. Dazu sollen für zentrale Merkmale jeweils elementare Fragestellungen beantwortet werden: Wie häufig ist das Merkmal in der Bevölkerung? Wie verteilt es sich zwischen verschiedenen Bevölkerungsgruppen? Welche Entwicklungen zeigen Häufigkeiten und Verteilungen langfristig? Beim Vergleich verschiedener Merkmale ist die Frage zu beantworten, inwiefern sich ähnliche oder divergierende Entwicklungen zeigen.

### Indikatoren und Datengrundlagen

Damit regelmäßige Bestandsaufnahmen gesundheitliche Entwicklungen umfassend beschreiben können, bilden sie möglichst das gesamte MHS-Indikatorenset ab [8]. Es umfasst sowohl positive psychische Gesundheit als auch Psychopathologie und bietet so Ansatzpunkte für Maßnahmen der Gesundheitsförderung und Prävention, Kuration und Rehabilitation [24].

Auf diese Weise können zeitliche Entwicklungen verschiedener Indikatoren vergleichend betrachtet und Hypothesen zu ihrem Zusammenspiel generiert werden. Mögliche Zunahmen psychischer Symptome können so vor dem Hintergrund von Entwicklungen bei Risiko- und Schutzfaktoren gedeutet werden. Veränderungen der Häufigkeit psychischer Störungen in der Bevölkerung können besser verstanden werden, wenn gleichzeitige Veränderungen der Sterblichkeit berücksichtigt werden, da diese die Anzahl prävalenter Fälle beeinflusst [25]. Auch eine Einschätzung des Gesamteffektes (Public Health Impact) der Gesundheitsversorgung oder anderer Maßnahmen erfordert eine vergleichende Betrachtung der Entwicklungen sowohl von Morbidität und Behandlungsbedarfen als auch von Behandlungsangebot, -nachfrage und -qualität [26].

**Figure 1:**
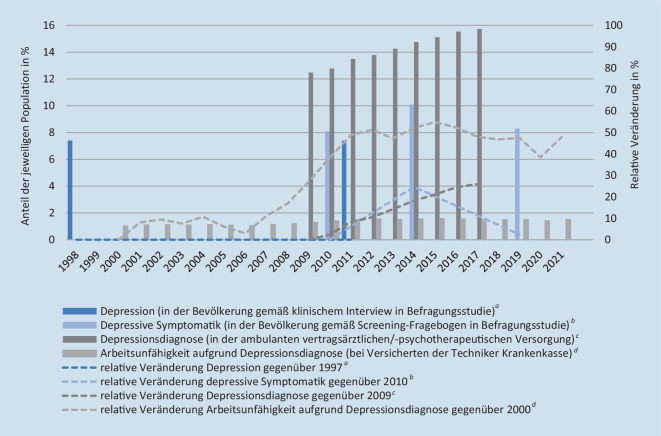

Wie die Indikatoren selbst soll auch deren Datengrundlage umfassend sein und verschiedene Informationsquellen einbeziehen. Ein Teil der Merkmale muss aus Perspektive des Individuums eingeschätzt werden und erfordert repräsentative Daten aus Befragungsstudien. Andere Indikatoren können in Routinedaten des Versorgungsgeschehens oder der amtlichen Statistik abgebildet werden. Eine Ausnahme stellt die Prävalenz psychischer Störungen dar, die sowohl in Bevölkerungsstudien als auch Routinedaten vergleichend bewertet (trianguliert) werden soll (siehe Beispiel in Abb. 1).

In welchen Zeitabständen derartige Bestandsaufnahmen aktualisiert werden, sollte primär von Informationsbedarfen für die Steuerung von PMH-Maßnahmen abhängen. Zugleich begrenzen finanzielle und personelle Anforderungen eines Surveillance-Systems, wie häufig neue Daten erhoben oder ausgewertet werden können. Aktuell wird in der Abteilung Epidemiologie und Gesundheitsmonitoring des RKI ein Gesundheitspanel aufgebaut, das ressourcenschonende und stabile Erhebungen in kontinuierlichen Abständen und mit schneller Datenverfügbarkeit ermöglichen kann.

Entscheidend für Surveillance ist, dass Datengrundlagen Vergleiche über die Zeit erlauben. Abb. 1 zeigt, inwiefern diese für depressive Störungen zum aktuellen Stand noch begrenzt sind. Will man die Entwicklung der Häufigkeit von Depression in der Bevölkerung mit der Entwicklung ihrer Versorgungslage und Krankheitslast vergleichen, ist dies gegenwärtig erschwert, u. a. durch abweichende Beobachtungszeiträume und Stichproben bzw. Bezugspopulationen sowie wechselnde Erhebungsmodi. Einige dieser Herausforderungen könnten durch entsprechende Studienplanung und kassen- und sektorenübergreifend zusammengeführte Routinedaten zukünftig überwunden werden.

### Auswertung, Berichterstattung und Interpretation

Ziel der Datenauswertungen für PHS sind vorrangig vergleichbare deskriptive Schätzungen für bestimmte Beobachtungszeiträume [32], damit zeitliche Trends in der Gesamtbevölkerung abgebildet werden können. Zusätzlich sollte dargestellt werden, wie sich Merkmale zwischen verschiedenen Bevölkerungsgruppen (nach Alter, Geschlecht, Region, Bildung, Migrationsstatus etc.) unterscheiden, um so Risikogruppen zu identifizieren und die Entwicklung gesundheitlicher Ungleichheit im Zeitverlauf zu beobachten. Darüber hinaus kann es in Anknüpfung an die Populationsstrategie von Geoffrey Rose [33] für PMH besonders wertvoll sein, nicht nur die Entwicklung der Häufigkeit dichotomisierter Merkmale zu beschreiben, sondern auch die der Verteilung verschiedener dimensionaler Maße der psychischen Gesundheit in der Bevölkerung [34].

Zu relevanten komplexen Auswertungsmethoden zählen Trendanalysen, mit denen zeitliche Veränderungen inferenzstatistisch bewertet werden können [32]. Demografische Entwicklungen sollten dabei kontrolliert werden, weil sie Prävalenztrends psychischer Störungen beeinflussen können [35]. Bei hinreichender Datenlage können Methoden der Zeitreihenanalyse genutzt werden, um z. B. in Public-Health-Impact-Analysen Veränderungen nach Maßnahmen oder kollektiven Ereignissen zu untersuchen [36]. Zeitreihen können auch herangezogen werden, um Alters‑, Perioden- und Kohorteneffekte voneinander abzugrenzen [37] oder Vorhersagen zu treffen [38].

Zur Kommunikation deskriptiver Ergebnisse bieten sich Onlineplattformen an [39, 40]. Für eine vertiefte Interpretation und Diskussion von Trends verschiedener Indikatoren eignen sich dagegen Berichte und Fachpublikationen [25, 41]. Anders als bei unten dargestellten MHS-Strategien ist eine zeitnahe Kommunikation aktueller Ergebnisse weniger dringlich, da neue Daten nicht engmaschig, sondern tendenziell im Abstand einiger Jahre erhoben werden.

Im Vordergrund der Interpretation von Ergebnissen stehen Form und Uniformität zeitlicher Entwicklungen. In Bezug auf Abb. 1 lässt sich beispielsweise diskutieren, dass depressive Symptome oder Störungen in der Bevölkerung (eher) nicht zugenommen haben, während die Häufigkeiten ambulanter Depressionsdiagnosen und so begründeter Arbeitsunfähigkeit ansteigen. Auch wenn abweichende Beobachtungszeiträume und Referenzpopulationen Interpretationen erschweren, lässt sich als Hypothese formulieren, dass der Prävalenzanstieg im Versorgungsgeschehen vermutlich vorrangig ein geändertes Inanspruchnahme- und/oder Diagnoseverhalten zum Ausdruck bringt und weniger eine Morbiditätszunahme.

### Grenzen und Entwicklungspotenziale

Bislang liegen nur für wenige der konsentierten MHS-Indikatoren Ergebnisse vor, die einen Ausgangspunkt für den Aufbau von Zeitreihen bieten. Insofern kann momentan vorrangig der Bedarf regelmäßiger umfassender Bestandsaufnahmen dargestellt und ihr Aufbau geplant werden.

Bei diesem Aufbau können verschiedene Entwicklungspotenziale beachtet werden: Informationen zu verschiedenen Indikatoren sollten möglichst für dieselben Personen vorliegen, z. B. um ökologische Fehlschlüsse zu vermeiden. Inwiefern Messinstrumente zwischen unterschiedlichen Gruppen und Messzeitpunkten vergleichbar sind, muss durch Messinvarianzanalysen überprüft werden. Um Aussagen zur Allgemeinbevölkerung treffen zu können, müssen alle relevanten gesellschaftlichen Gruppen in den genutzten Daten abgebildet werden. Insbesondere Menschen mit Migrationshintergrund sollten als große und wachsende Bevölkerungsgruppe mit speziellen Risiko- und Schutzfaktoren besser in Bevölkerungssurveys eingebunden werden [42], wozu vielversprechende methodische Ansätze vorliegen [43–45].

Grundsätzlich bleibt zu berücksichtigen, dass der Erhalt psychischer Gesundheit und die Pathogenese psychischer Störungen außerordentlich komplex sind. MHS kann diese Komplexität nicht vollständig erfassen, aber Grundzüge eines Gesamtbildes skizzieren, das langfristig wesentliche Dynamiken abbildet.

## Hochfrequente Beobachtung: Aktuelle Veränderungen und Frühwarnsystem

### Ausgangslage

Vor dem Hintergrund des akuten Informationsbedarfs in der dynamischen COVID-19-Pandemie [6] begann an mehreren Public-Health-Instituten weltweit der Aufbau einer zeitlich engmaschigen Surveillance der psychischen Gesundheit, meist basierend auf wöchentlichen oder monatlichen Kennzahlen zu ausgewählten Indikatoren (z. B. Frankreich [46], Vereinigtes Königreich [47] und Vereinigte Staaten von Amerika [48]). Parallel wurde auch in der MHS am RKI ein solcher Ansatz entwickelt, der im Bereich PMH neu und der Überwachung von Infektionserkrankungen entlehnt ist. Die hochfrequente Beobachtung soll im Sinne eines „Frühwarnsystems“ Politik und Gesundheitsversorgung dazu befähigen, umgehend auf aktuelle Veränderungen zu reagieren.

### Ziele und Fragestellungen

Ziel der engmaschigen Überwachung der psychischen Gesundheit ist es, Entwicklungen kontinuierlich und mit hoher zeitlicher Auflösung zu beobachten. *Primär* sollen aktuelle Trends verfolgt und Veränderungen frühzeitig erkannt werden. *Sekundär* können längerfristige zeitliche Verläufe beschrieben werden. Für eine Auswahl von Merkmalen der psychischen Gesundheit der Bevölkerung sind zu diesem Zweck 2 Fragen zu beantworten. *Primär: *Deuten jüngste Entwicklungen vor dem Hintergrund des Gesamtverlaufs aktuell auf eine Verschlechterung, Verbesserung oder Stabilität der psychischen Gesundheit in der Bevölkerung und in verschiedenen Bevölkerungsgruppen hin? *Sekundär:* Welche Entwicklungen zeigen die Merkmale über die Zeit, auch mit Blick auf mögliche zeitliche Koinzidenzen mit kollektiven Ereignissen oder (gesundheits‑)politischen Interventionen?

### Indikatoren und Datengrundlagen

Die hochfrequente Erhebung und Auswertung eines umfänglichen Indikatorensets sind nicht umsetzbar. Die Erfassung eines Gesamtbildes ist auch nicht das Ziel dieser Strategie, sondern die Entdeckung von Signalen von Veränderung.

Werden zu diesem Zweck wenige Indikatoren ausgewählt, sollte berücksichtigt werden, dass möglichst beide Kontinuen psychischer Gesundheit (Psychopathologie und positive psychische Gesundheit [24]) Beachtung finden und zur Früherkennung psychopathologischer Entwicklungen potenziell prodromale Belastungsanzeichen erfasst werden [49]. Indikatoren, die veränderte Versorgungsbedarfe oder Krankheitslast früh anzeigen, unterstützen die Einschätzung der Dringlichkeit von Reaktionen auf Surveillance-Ergebnisse.

In der COVID-19-Pandemie wurde im Zusammenhang mit Kontaktbeschränkungen auch die Surveillance bestimmter sozialer Determinanten psychischer Gesundheit priorisiert [6]. Ultrakurze Erhebungsinventare zur Messung der Indikatoren erlauben eine ökonomische Umsetzung von Primärdatenerhebungen. Entsprechend werden am RKI aktuell 6 Indikatoren mit sehr kurzen Fragenbögen von jeweils 1–3 Items hochfrequent erhoben: depressive Symptome [50, 51], Symptome von Angststörungen [52], subjektive psychische Gesundheit [53], Behandlungsbedarf, Einsamkeit [54] und soziale Unterstützung [55].

Voraussetzung für eine aussagekräftige engmaschige Surveillance ist die kontinuierliche und möglichst lückenlose Datenerhebung und/oder -verfügbarkeit ohne methodische Veränderungen. Außerdem müssen eine ausreichende Stichprobengröße sowie die Repräsentativität der Daten im kleinsten zu betrachtenden Zeitintervall gegeben sein. Die Frequenz der Surveillance ist also durch bestimmte Eigenschaften der Daten begrenzt. Die zeitliche Auflösung der Beobachtung psychischer Gesundheit – tage-, wochen-, monats- oder quartalsweise – sollte sich grundsätzlich an den Informationsbedarfen orientieren, die an eine MHS gerichtet werden. Die Veränderlichkeit der Indikatoren bzw. die Veränderungssensitivität der Erhebungsinstrumente muss mit Blick auf die zeitliche Auflösung zunächst konzeptuell und empirisch geprüft werden, um die Veränderungssensitivität der Surveillance als Ganzes zu optimieren [56].

### Auswertung, Berichterstattung und Interpretation

**Figure 2:**
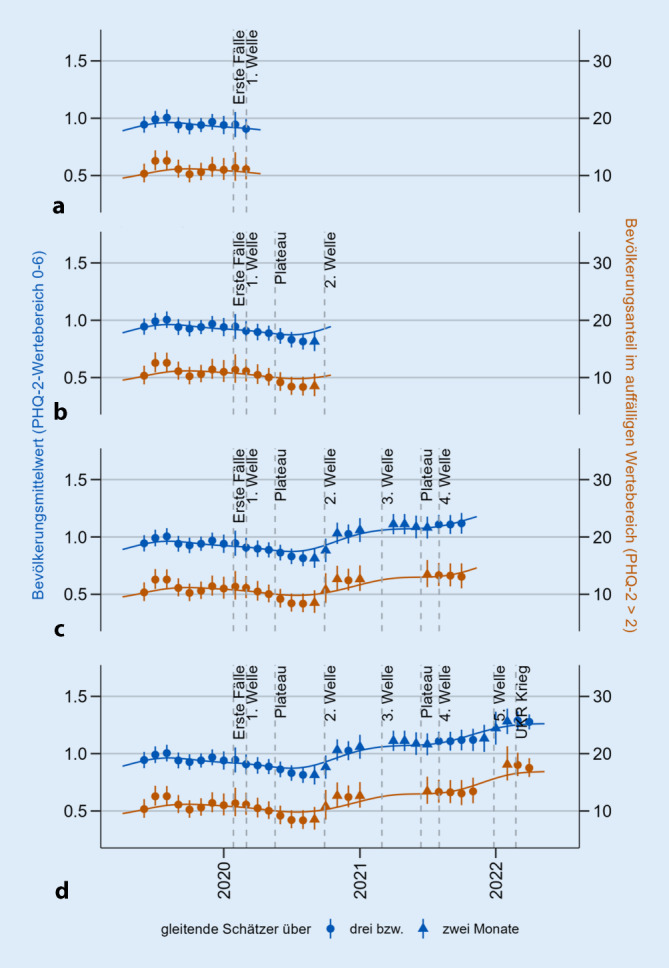

Besonderheiten der Datenauswertung in der hochfrequenten Surveillance lassen sich anhand eines Beispiels aus der laufenden MHS am RKI illustrieren: der Überwachung von depressiven Symptomen gemessen mit dem Ultra-Kurz-Screener Patient Health Questionnaire‑2 (PHQ‑2, Abb. 2, siehe auch [57]). Die PHQ-2-Ergebnisse werden zum einen dichotom nach validiertem Cut-Off ausgewertet, um den Bevölkerungsanteil im auffälligen Wertebereich zu ermitteln [51]. Zum anderen wird zur Unterstützung der Früherkennungsfunktion ebenfalls die dimensionale Betrachtungsweise über Bevölkerungsmittelwerte herangezogen, um Belastungsanzeichen im gesamten Wertebereich zu erfassen.

Angesichts des hohen Aktualitätsanspruchs erfolgt die Datenverarbeitung bis hin zum grafischen Output durch eine automatisierte Pipeline. Um trotz limitierter Stichprobengröße eine möglichst hohe zeitliche Auflösung zu erreichen, werden monatlich gleitendende 3‑Monats-Schätzer berechnet. Monatliche Schätzungen beruhen dadurch auf ausreichend großen Substichproben. Außerdem werden Zeitreihen durch gleitende Schätzungen [32] sowie durch zusätzliche Glättungskurven [60] um stichprobenbedingte Schwankungen bereinigt. Die Zeitreihen werden ebenfalls stratifiziert nach Geschlecht, Alter und Bildung berechnet, damit gruppenspezifische Entwicklungen beobachtet werden können.

Für das primäre Ziel fortlaufender, frühzeitiger Erkennung von Veränderung werden gleitende 3‑Monats-Schätzer samt Konfidenzintervallen und Glättungskurven nach jeder Aktualisierung durch neue Daten zunächst visuell untersucht. Als mögliche Veränderungen in einem Indikator werden alle Entwicklungen gewertet, die 1) über mehrere 3‑Monats-Schätzer weiterverlaufen (Abb. 2b) oder anhalten (Abb. 2c) und 2) im Gesamtverlauf auffällig sind. Zusätzlich können statistische Vergleiche zwischen den jüngsten Schätzungen und Schätzungen aus passenden Vergleichszeitfenstern durchgeführt werden. Je länger die Zeitreihe, desto besser können Entwicklungen als (weitere) Verbesserungen, (weitere) Verschlechterungen (Abb. 2d) oder Stabilität (nach Verschlechterung (Abb. 2c) oder Verbesserung) eingeordnet werden. Insofern sind fortlaufende Erhebungen notwendig, um Entwicklungen während Krisen einschätzen zu können. Zeigen sich Hinweise auf eine bestimmte Veränderung in mehreren Indikatoren, erhöht dies die Evidenz.

Welche Zeitfenster in der laufenden Surveillance oder zur Beschreibung längerer Verläufe verglichen werden, ergibt sich einerseits datengetrieben aus beobachteten Entwicklungen. Andererseits können Vergleiche top-down bestimmt werden, etwa bei zu untersuchenden zeitlichen Koinzidenzen mit gesellschaftlichen Entwicklungen (z. B. prä- versus peripandemisch [57]) oder für Public-Health-Impact-Analysen. Zur Untersuchung längerer Verläufe könnten auch komplexere Ansätze aus dem Bereich Trend- und Zeitreihenanalysen Anwendung finden (siehe z. B. [61]).

Damit die engmaschige Surveillance eine Frühwarnfunktion bieten kann, sollte die Berichterstattung mit geringstmöglichem Zeitverzug erfolgen und sich in ihrer Taktung an der Frequenz neuer Schätzungen orientieren [62]. Aus diesem Grund wird die Kommunikation von Ergebnissen per Dashboard angestrebt [46–48]. Die Berichterstattung zur Betrachtung längerer Zeitreihen erfolgt über Fachpublikationen [57] oder ausführlichere Berichte. Das Bundesministerium für Gesundheit (BMG) wird regelmäßig über aktuelle Entwicklungen informiert.

### Grenzen und Entwicklungspotenziale

Als neue MHS-Strategie befindet sich die hochfrequente Beobachtung insgesamt noch in Entwicklung. Bei länger und komplexer werdenden Zeitreihen könnten perspektivisch modellbasierte Ansätze zur Detektion von Veränderungen [63] die Interpretation unterstützen.

Es steht außerdem aus, gestützt durch Empirie und gemeinsam mit rezipierenden Akteuren für die jeweiligen Indikatoren zu bestimmen, welche Veränderungen für Public (Health) Policy bedeutsam sind. Zudem wird die Möglichkeit der Ausweitung auf Routinedaten geprüft, aktuell insbesondere die Eignung von Notaufnahmedaten für eine hochfrequente Beobachtung.

In Krisenzeiten birgt eine hochfrequente Surveillance weniger Indikatoren mit einem zeitnahen und fortlaufenden Reporting besondere Potenziale. Da eine adäquate Einordnung der sich in Krisenzeiten zeigenden Verläufe die vorherige kontinuierliche Beobachtung voraussetzt, soll die hochfrequente Surveillance neben ihrer allgemeinen Funktion als Frühwarnsystem zu jeder Zeit auch im Sinne der Krisenbereitschaft Teil der regulären MHS-Praxis werden.

## Kontinuierliches Literaturreview: Forschungstätigkeit, -stand und -bedarfe

### Ausgangslage

In der COVID-19-Pandemie wurde mit ungewohnt hohem Tempo und Umfang Evidenz zur Entwicklung der psychischen Gesundheit der Bevölkerung generiert. Entsprechend entstand rasch der Bedarf, Forschungsergebnisse in Überblicksarbeiten zu sondieren und zusammenzufassen, um sie politischen Akteuren zur Verfügung zu stellen [64].

In verschiedenen Ländern führten Gesundheitsbehörden während der Pandemie Recherchen und Evidenzsynthesen ein, um die nationale Studienlage zur psychischen Gesundheit zu bewerten, zum Beispiel im Vereinigten Königreich [65] und in der Schweiz [66]. Für Deutschland legte das RKI entsprechende Übersichtsarbeiten vor, die Ergebnisse zur erwachsenen Bevölkerung [67] sowie zu Kindern und Jugendlichen [68, 69] aufbereiten. Allerdings blieb dieser Informationsstand nicht lange aktuell, da beständig neue Ergebnisse veröffentlicht wurden.

Vor diesem Hintergrund wurde als dritte MHS-Strategie ein kontinuierliches Literaturreview aufgesetzt. Es führt den Ansatz der publizierten Arbeiten fort, die als Rapid-Reviews [67, 69] durch methodische Vereinfachungen schneller erstellt werden können als systematische Reviews [70]. Die fortlaufende Aktualisierung lehnt sich zudem an die Methode von Living-Systematic-Reviews für stetig wachsende und heterogene Studienlagen an [71], zu der aktuelle Beispiele für psychische Gesundheit vorliegen [72, 73].

### Ziele und Fragestellungen

Ziel des kontinuierlichen Literaturreviews ist die regelmäßige Erfassung des aktuellen Forschungsstandes zur Entwicklung der psychischen Gesundheit der Bevölkerung. In seiner aktuellen Konzeption ist das Review auf gesundheitliche Trends während der COVID-19-Pandemie bezogen. Es ermöglicht eine zusammenfassende Bewertung der Evidenzlage, eine Einordnung von Einzelbefunden sowie die Identifikation gegenwärtig prioritärer Forschungsbedarfe.

Die kontinuierliche Erfassung veröffentlichter Forschungsergebnisse soll folgende Fragen beantworten: Für welche Beobachtungszeiträume und Indikatoren liegen veröffentlichte Ergebnisse zur Entwicklung psychischer Gesundheit in der Allgemeinbevölkerung während der COVID-19-Pandemie vor? Wie verlässlich sind Daten und Ergebnisse? Wie ist die vorliegende Evidenz zusammenfassend zu bewerten?

### Indikatoren und Datengrundlagen

Die Suchstrategie zielt auf Publikationen zu Veränderungen der psychischen Gesundheit während der Pandemie, ohne diese durch präzisere Suchbegriffe einzugrenzen. Die in den gefundenen Arbeiten untersuchten Outcomes lassen sich den Themen positive psychische Gesundheit, psychische Belastungen, aktuelle Symptomatik psychischer Störungen sowie Versorgungslage/Mortalität zuordnen. Welche spezifischen Konstrukte erhoben und wie sie operationalisiert wurden, unterscheidet sich zwischen den eingeschlossenen Studien zum Teil stark [67].

Die Suchgrundlage umfasst einschlägige Datenbanken sowie Preprint-Server, um möglichst aktuelle Ergebnisse einzubeziehen [67]. Da nicht alle für MHS relevanten Ergebnisse in Fachjournalen veröffentlich werden, wird zusätzlich eine Handrecherche durchgeführt. Aktuell erfolgt die Aktualisierung in monatlichem Turnus. Ein- und Ausschlusskriterien des Reviews orientieren sich an übergeordneten MHS-Zielen, zum Beispiel der Zielpopulation „Allgemeinbevölkerung“ und dem Bericht von zeitlichen Entwicklungen.

### Auswertung, Berichterstattung und Interpretation

**Figure 3:**
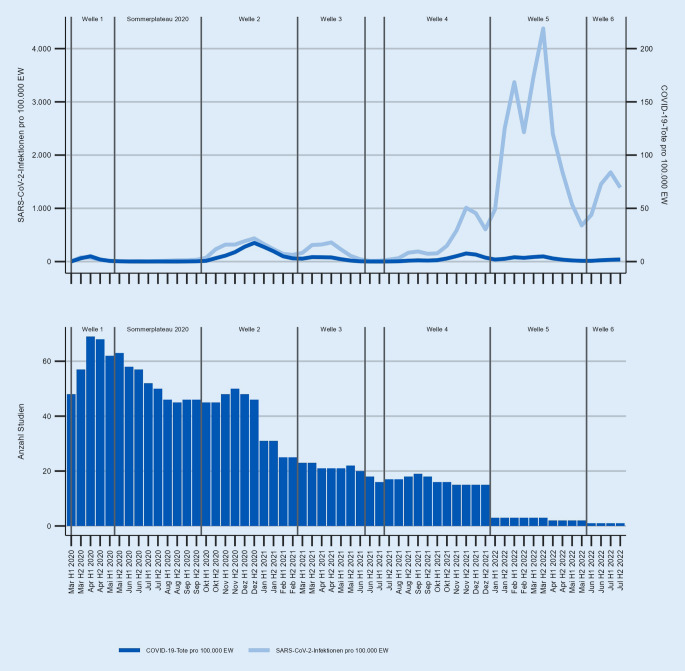

Um für MHS genutzt zu werden, setzt das kontinuierliche Literaturreview folgende Schwerpunkte bei der Datenauswertung:

(1) Es wird eingeschätzt, wie verlässlich Aussagen zu Trends in der Allgemeinbevölkerung auf Basis des Forschungsstandes getroffen werden können. Dazu wird u. a. bewertet, wie verzerrungsanfällig Ergebnisse aufgrund von Stichprobenziehung und Veränderungsmessung sind [67]. (2) Die Beobachtungszeiträume der eingeschlossenen Studien werden ausgewertet. Wie Abb. 3 zeigt, ist so erkennbar, für welche Zeiträume der COVID-19-Pandemie Evidenz vorliegt beziehungsweise (noch) fehlt. (3) Indem die beforschten Konstrukte und deren Operationalisierungen systematisiert werden, zeigt sich, wie viel Evidenz für welche Themen vorliegt und wie heterogen diese ist. (4) Bei wenigen oder inhaltlich heterogenen Ergebnissen werden beschreibende Zusammenfassungen (narrative Evidenzsynthesen) zur Bewertung des Forschungsstandes genutzt [73]. So wird aktuell je Pandemiephase ausgewertet, wie viele Publikationen einen Anstieg, einen Rückgang oder unveränderte Outcomes berichten, um allgemeine Tendenzen zeitlicher Trends psychischer Gesundheit zu identifizieren.

Um den Anforderungen an zeitnahes Reporting gerecht zu werden, eignen sich Onlineplattformen oder Dashboards zur Ergebnisdissemination [74, 75]. Gegenwärtige Adressaten der Berichterstattung des kontinuierlichen MHS-Literaturreviews sind Akteure des Öffentlichen Gesundheitsdienstes [76–78] sowie die Fachöffentlichkeit [79]. Eine nächste Fachpublikation ist in Vorbereitung. Das BMG wird monatlich über die aktuelle Evidenzlage informiert.

Einen besonderen Nutzen bringt das kontinuierliche Literaturreview, wenn es mit der Strategie hochfrequenter Datenanalysen (siehe Kapitel „Hochfrequente Beobachtung“) kombiniert wird. Einzelergebnisse können so in den Forschungsstand eingeordnet werden und Evidenzlücken Eingang in die Studienplanung finden (vgl. auch [74]).

### Grenzen und Entwicklungspotenziale

Als MHS-Strategie weist das kontinuierliche Literaturreview verschiedene Grenzen und Entwicklungspotenziale auf: Die Recherche kann durch Methoden der Automatisierung noch engmaschiger aktualisiert werden [73]. Bei hinreichender Anzahl und Vergleichbarkeit von Studien können Evidenzsynthesen durch Metaanalysen ergänzt werden, wofür ebenfalls Aktualisierungsansätze vorliegen [80].

Eine Herausforderung für den Arbeitsprozess liegt darin, dass die Anzahl neuer Publikationen nie abschätzbar ist und damit Flexibilität erfordert. Auf lange Sicht kann sich die Frequenz der Recherche auch nach dem Publikationsaufkommen richten. Die gegenwärtig auf die COVID-19-Pandemie ausgerichtete Suchstrategie bedarf der Aktualisierung, sobald die Pandemie abklingt bzw. andere kollektive Belastungsfaktoren für die psychische Gesundheit aufkommen. Damit stellt die Ausweitung der Fragestellung auf zeitliche Entwicklung der psychischen Gesundheit allgemein (statt „während der COVID-19-Pandemie“) eine langfristig notwendige methodische Weiterentwicklung dieser MHS-Strategie dar.

## Diskussion

Beim Aufbau der MHS für Deutschland wurden am RKI 3 Strategien zur Beobachtung der psychischen Gesundheit der Bevölkerung entwickelt (Tab. 1): Regelmäßige Bestandsaufnahmen haben die systematische Beobachtung von langfristigen Veränderungen zum Ziel, um ein umfassendes Gesamtbild psychischer Gesundheit zu skizzieren. Hierzu können bisher erst wenige Daten genutzt werden. Die Strategien der hochfrequenten Beobachtung und des kontinuierlichen Literaturreviews zielen darauf ab, aktuelle Entwicklungen frühzeitig zu erkennen. Genutzt werden dazu aktuell engmaschig erhobene Befragungsdaten sowie monatliche Literaturübersichten der aktuellen Evidenzlage.

### Funktionen von Mental Health Surveillance

Die 3 skizzierten Strategien konnten bislang unterschiedlich weit entwickelt werden. Bereits jetzt und noch mehr bei vollem Ausbau einer MHS können sie gemeinsam dazu beitragen, dass Kernfunktionen eines PHS-Systems erfüllt werden [4]:

Die Bedeutung psychischer Gesundheit für die Bevölkerungsgesundheit kann quantifiziert werden. Die Verteilung von positiver psychischer Gesundheit sowie die Verbreitung psychischer Störungen können regelmäßig dokumentiert werden. Negative Entwicklungen können bei engmaschiger Beobachtung frühzeitig erkannt werden. Hypothesen zum Zusammenspiel verschiedener Indikatoren sowie zu Einflüssen des Zeitgeschehens können generiert werden. Insgesamt können somit Forschungs- und Handlungsbedarfe benannt werden. Auch wenn MHS die Wirksamkeit einzelner Maßnahmen nicht nachweisen kann, erlaubt sie doch zu beobachten, ob ein intendierter Public Health Impact auf Bevölkerungsebene erzielt wurde. Zusätzlich könnten (unter bestimmten empirischen Voraussetzungen) Prognosen zu zukünftigen Entwicklungen im Feld psychischer Gesundheit formuliert werden [38].

### Chancen für verbesserte Public-Mental-Health-Praxis

Wenn MHS die oben genannten Funktionen erfüllt, kann sie auf verschiedenen Ebenen zur Verbesserung der gesundheitlichen Lage beitragen [41, 56]:

1. Awareness: Durch Informationen über aktuelle Entwicklungen können Akteure aus Politik und Praxis entsprechend wachsam sein. Sie können Bedarfe von Prävention und Versorgung besser erkennen, auch im direkten Kontakt mit Betroffenen.
2. Agenda-Setting: Ergebnisse von MHS können anzeigen, inwiefern psychische Gesundheit in Public-Health-Programmen und -Forschung priorisiert werden sollte.
3. Advocacy: Indem MHS individuelles Erleben aggregiert erfasst, Wohlbefinden gleichermaßen wie Leiden, können Bedarfe Einzelner zum Thema des Diskurses zur Bevölkerungsgesundheit werden. So können auch Personengruppen vertreten werden, deren Problemlagen anderweitig wenig Gehör finden.

Insgesamt kann MHS prinzipiell die Erreichung (gesundheits-)politischer Ziele im Feld psychischer Gesundheit monitoren und unterstützen. Allerdings ist der Gesundheitszieleprozess in Deutschland inhaltlich noch stark begrenzt (Bsp. Gesundheitsziel Depression [81]) und bietet keine expliziten messbaren Ziele für das gesamte Feld psychischer Gesundheit (wie z. B. in Australien [82]). Darüber hinaus müssen rezipierende Akteure die Nutzung von Surveillance-Informationen erst (kennen-)lernen (vgl. auch [83]).

Idealerweise würde fortlaufende Surveillance im Austausch zwischen berichterstattenden und rezipierenden Akteuren gestaltet werden, sodass Surveillance-Prozesse und Response-Prozesse optimal ineinandergreifen könnten [84]. Dabei hängen die Informationsbedarfe von Akteuren sowohl von Zielsetzungen als auch vom verfügbaren Repertoire an Interventionen ab. Die COVID-19-Pandemie hat gezeigt, dass grundsätzlich Maßnahmen der Gesundheitspolitik für Förderung und Schutz der psychischen Gesundheit zur Verfügung stehen, die auch ad hoc eingesetzt werden können [85–87].

Im Sinne eines Mental-Health-in-All-Policies-Ansatzes können auch Maßnahmen anderer Ressorts auf die psychische Gesundheit Einfluss nehmen, wobei ihre Bedeutung in der Entscheidungsfindung stets Gegenstand normativer Abwägungen bleibt. Als deren Grundlage forderte der Deutsche Ethikrat im Zusammenhang mit der COVID-19-Pandemie eine staatliche „Verpflichtung zur Wissensgenerierung“ auch zur psychischen Gesundheit [88].

Eine effektive Steuerung von Maßnahmen ist erst dann möglich, wenn eine Informationsgrundlage geschaffen wird, die nicht nur zeitlich, sondern auch räumlich angemessen aufgelöst ist, da Maßnahmen oft lokal umgesetzt werden. Jede weitere Stratifizierung von Ergebnissen (z. B. nach Migrationsstatus) kann dazu beitragen, dass vulnerable und resiliente Gruppen oder Zielgruppen von Interventionen genauer erkannt werden können.

### Fazit

Mental Health Surveillance (MHS) kann verschiedene Strategien zur Beobachtung der psychischen Gesundheit der Bevölkerung einsetzen, die optimalerweise kombiniert werden. So kann zur Vorbereitung auf mögliche gesundheitliche Krisen beigetragen werden (Preparedness), eine effektive Reaktion auf Krisen (Response) unterstützt und die Erreichung gesundheitlicher Ziele (auch außerhalb von Krisen) überwacht werden.

Eine Evaluation des Systems sollte langfristig zeigen, ob die skizzierten Strategien nachweislich nützlich sind [56]. Der Betrieb einer effektiven MHS hat Implikationen für die gesundheitliche Lage: Anstrengungen zur Verbesserung der psychischen Bevölkerungsgesundheit sollten nicht von lückenhafter Evidenz unterminiert werden. Auch Erfolge dieser Anstrengungen können erst durch MHS sichtbar gemacht werden.
